# The Macklin Effect Following Trauma: A Case Report of Concurrent Pneumothorax, Pneumoperitoneum, Pneumomediastinum, and Pneumoretroperitoneum in an Intubated Young Adult

**DOI:** 10.7759/cureus.74901

**Published:** 2024-12-01

**Authors:** Ahmad A Alrahmani, Fayez G Aldarsouni, Lujain S Alwasel, Faisal S AlGhamdi, Sara W Abdelhamid, Khaled Twier

**Affiliations:** 1 Department of General Surgery, East Jeddah Hospital, Jeddah, SAU; 2 Department of Surgery, King Saud University Medical City, Riyadh, SAU; 3 Department of Trauma Surgery, King Saud Medical City, Riyadh, SAU; 4 Department of General Surgery, Prince Sultan Military Medical City, Riyadh, SAU; 5 College of Medicine, Alfaisal University, Riyadh, SAU

**Keywords:** macklin effect, pneumomediastinum, pneumoperitoneum, pneumoretroperitoneum, pneumothorax, subcutaneous emphysema, thoracic injuries, trauma

## Abstract

Pneumomediastinum, often a silent yet disruptive force in the context of trauma, complicates clinical decision-making, particularly when it is accompanied by pneumothorax, pneumoperitoneum, and pneumoretroperitoneum. The Macklin effect, where air dissects along tissue planes following alveolar rupture, frequently underpins these findings, adding layers to the diagnostic puzzle.

In this case, an 18-year-old male involved in a high-speed vehicle collision was transferred to our trauma center intubated and sedated. Initial imaging painted a daunting picture: pneumomediastinum, a sizable left-sided pneumothorax, and extensive subcutaneous emphysema. Further, a whole-body computed tomography scan revealed the additional complications of pneumoperitoneum and pneumoretroperitoneum. Despite the concerning radiographic findings, endoscopic evaluations found no evidence of esophageal or bronchial injury. Management was conservative, including chest tube placement and monitoring, and resulted in a gradual resolution of symptoms. The patient's in-hospital course was uneventful, and he was discharged in stable condition without further complications.

The presence of pneumomediastinum with associated air in other compartments triggers a reflex to consider severe, life-threatening conditions like esophageal rupture. However, this case highlights the importance of differentiating between such critical injuries and less ominous causes like the Macklin effect. In trauma, the art lies in knowing when to intervene and when to trust the body's capacity to heal, supported by careful observation and conservative management.

## Introduction

Pneumomediastinum, characterized by air accumulation around mediastinal structures, is identified in approximately 10% of patients with severe blunt chest trauma [1]. The etiology of this condition can be multifaceted, encompassing tracheobronchial disruptions and, to a lesser extent, esophageal injuries [2]. Additional sources of pneumomediastinum may include the extension of emphysematous processes from the cervical and thoracic regions or as a consequence of retro-pneumoperitoneum, which serves as an indicator of hollow viscus injury [3,4]. The phenomenon of spontaneous pneumomediastinum (SPM) was first described by Charles Meklen in 1939, defined by the presence of mediastinal air without an obvious causative event [5]. The underlying pathophysiology is hypothesized to involve the seepage of air into peribronchovascular sheaths and intralobular septa within the mediastinum via the visceral pleura as a result of intrathoracic pressure elevation [6]. While Meklen's observation is commonly associated with blunt trauma, it also manifests in various clinical scenarios, such as neonatal respiratory distress syndrome, status asthmaticus, the use of positive pressure mechanical ventilation, epileptic seizures, and Boerhaave's syndrome [2,7].

Typically, the resolution of such injuries occurs spontaneously with conservative management that includes rest and analgesia. Nonetheless, in the context of trauma, the detection of pneumomediastinum may obfuscate the clinical picture by mimicking other pathologies, particularly esophageal perforation, bowel rupture, or tracheobronchial injury. This diagnostic ambiguity poses a challenge, as misdiagnosis can lead to unnecessary surgical explorations or, conversely, a missed injury [8].

The concurrent presentation of pneumomediastinum with pneumothorax and pneumoperitoneum is a rare triad [9]. This case report describes the presentation of a young adult who suffered a high-impact road traffic accident, was intubated, and presented with traumatic pneumomediastinum associated with pneumothorax and pneumoperitoneum, underscoring the critical differential diagnosis between esophageal rupture and SPM in such traumatic contexts.

## Case presentation

**Figure 1:**
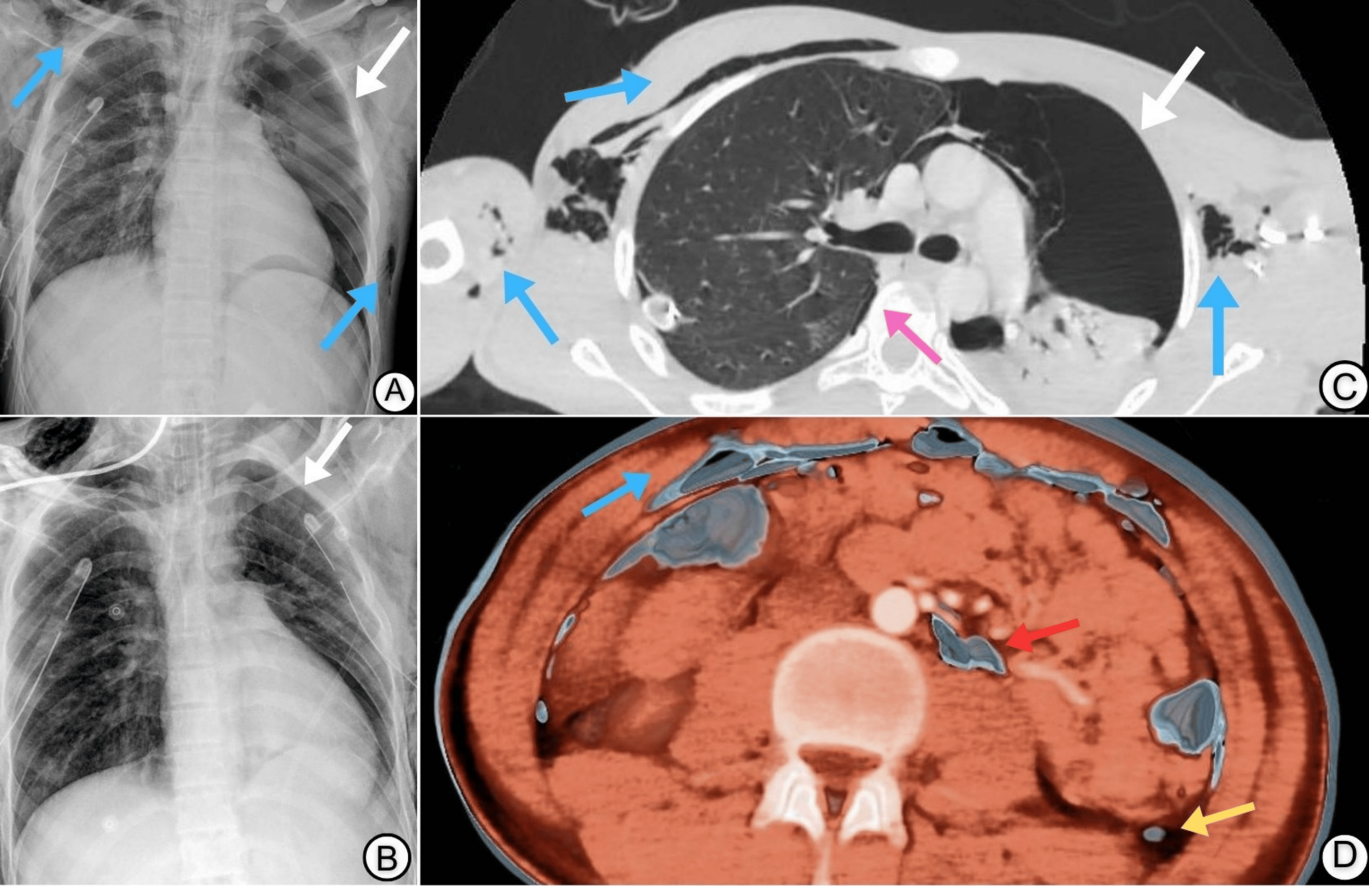
Chest and abdominal imaging findings in a trauma patient with the Macklin effect. The initial X-ray (A) showed a large left-sided pneumothorax (white arrows) with air accumulating in the pleural space, and subcutaneous emphysema (blue arrows). The follow-up X-ray (B) revealed the placement of a chest tube (white arrow), with partial re-expansion of the lung and some residual subcutaneous emphysema. (C) An axial computed tomography (CT) scan of the chest highlighted a significant left-sided pneumothorax (white arrow) with extensive air in the mediastinum (pink arrow) and subcutaneous tissues (blue arrows). (D) 3D reconstruction of the abdomen CT scan showed free air in the peritoneal cavity (red arrows) and retroperitoneal space (yellow arrow), indicating pneumoperitoneum and pneumoretroperitoneum, respectively, with air tracking from the thorax into the abdomen and previously noted subcutaneous emphysema (blue arrow).

**Figure 2:**
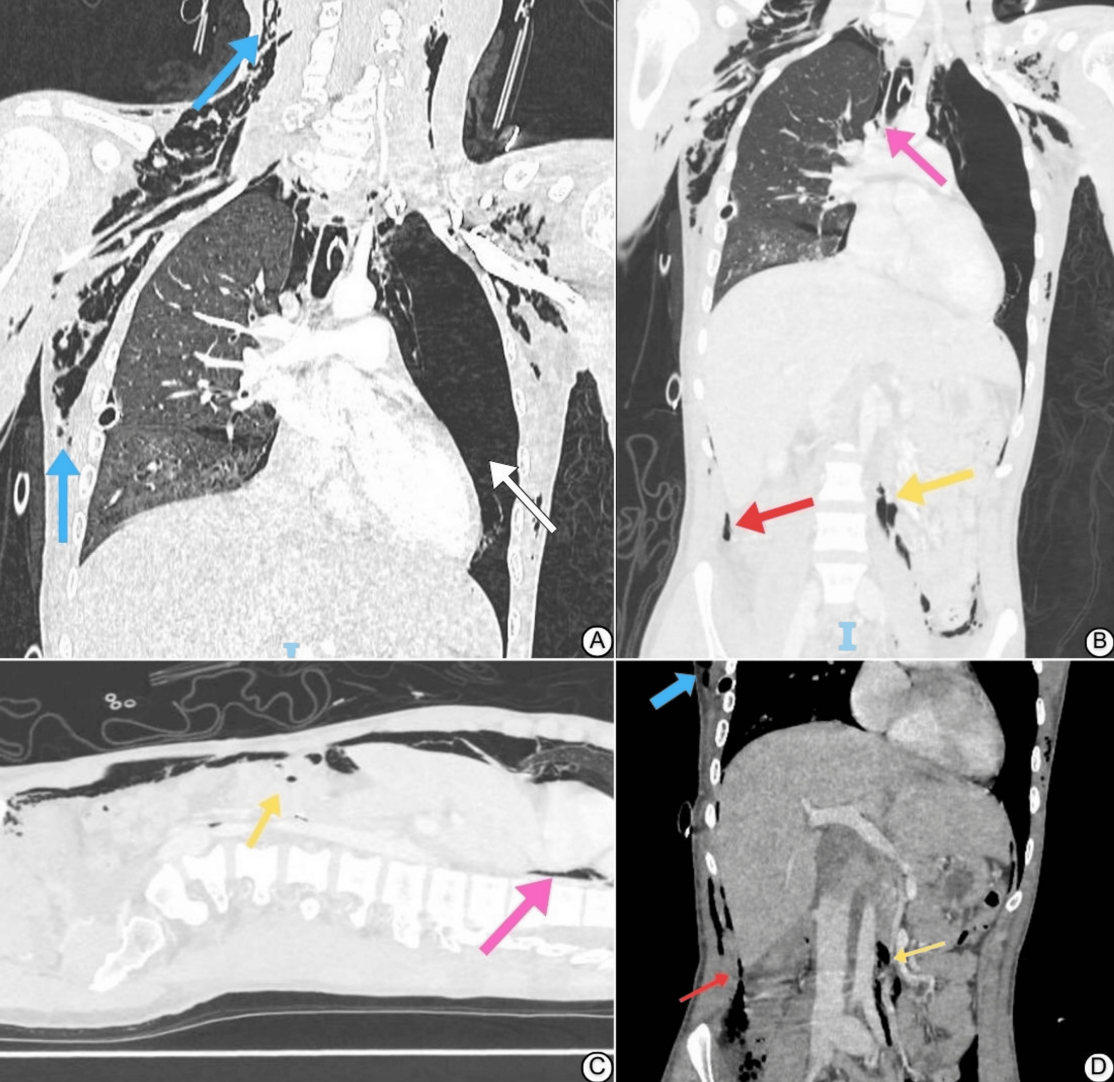
Coronal and sagittal computed tomography findings of the patient. (A) Coronal computed tomography chest view showing pneumothorax (white arrow) and extensive subcutaneous emphysema (blue arrows). (B) Coronal computed tomography chest view demonstrating pneumomediastinum (pink arrow) and pneumoperitoneum (red arrow). (C) Sagittal computed tomography view highlighting pneumoretroperitoneum (yellow arrow) and subcutaneous emphysema (blue arrow). (D) An abdominal window coronal computed tomography scan confirmed pneumoperitoneum (red arrow) and pneumoretroperitoneum (yellow arrow), with air tracking visible in the abdominal spaces, and previously noted subcutaneous emphysema (blue arrow).

An 18-year-old male was involved in a high-impact motor vehicle accident (MVA) with a lateral collision impacted by another vehicle traveling at a speed of 170 km/h. Initially managed at a rural hospital 500 km from our center, he was intubated due to an altered level of consciousness. After one day of high-setting mechanical ventilation, he was medevaced to our level 1 trauma center for further evaluation of a suspected esophageal injury after he had some signs of neck emphysema. Upon arrival, the patient was sedated, maintaining acceptable oxygen saturation on moderate mechanical ventilation settings (fraction of inspired oxygen (FiO2) = 50%; positive end-expiratory pressure (PEEP) = 8 cm H₂O), and was vitally stable (blood pressure = 110/60 mmHg; heart rate = 91 bpm). Physical examination revealed crepitus over the neck and anterior chest walls, decreased air entry upon auscultation on the left side of the chest, and a chest tube in place on the right side that was functional with no air leak. Chest X-ray showed pneumomediastinum and a left-sided pneumothorax (Figure 1A). Venous blood gas indicated alkalosis with low partial pressure of carbon dioxide (pCO2). A whole-body computed tomography (CT), part of the Advanced Trauma Life Support (ATLS) protocol (Figures 1C, 1D, 2), confirmed these findings and also showed extensive pneumomediastinum, a large left-sided pneumothorax, a small residual right lung pneumothorax, and minor abdominal and pelvic pneumoperitoneum and pneumoretroperitoneum. Significant subcutaneous and soft tissue emphysema was evident on the right shoulder, lower neck, and bilateral chest wall (Figure 2A). Management included the insertion of a left-sided intercostal drain for the pneumothorax (Figure 1B). Bronchoscopy and upper gastrointestinal endoscopy found no bronchial or esophageal injuries. Over the first week, chest tubes were removed after X-rays confirmed lung re-expansion and resolution of pneumomediastinum and surgical emphysema. The remainder of the inpatient stay was uneventful. After discharging the patient, he was followed up on a regular basis for six months, and he returned to his usual functional state of health. The patient consented to participate in this case report.

## Discussion

The phenomenon of pneumomediastinum represents a diagnostic dilemma stemming from its potential origination from both intrathoracic and extrathoracic sources. Complicating its diagnosis process, pneumomediastinum (blue arrows in Figures 1, 2) can mimic the radiographic picture of other high-risk presentations, as noted in the case we observed. Supporting literature suggests that SPM is prevalent among young males, potentially due to the decreased pliability of pulmonary interstitial tissues compared to the elderly population [7,8]. In older populations, this may confer a protective effect [10].

The prevalence of this condition remains ambiguous, likely underrecognized, owing to the non-specific nature of its clinical manifestations. In line with findings from Alemu et al., chest pain, dyspnea, and subcutaneous emphysema are frequently reported [7]. Our patient, who was intubated and sedated, presented a challenge in diagnosis due to the altered consciousness, even post extubation. Nevertheless, the presence of subcutaneous emphysema was evident. The employment of a full-body CT scan, adhering to ATLS, was instrumental in diagnosing the pneumomediastinum, as the radiographs prominently displayed pneumothorax.

The mechanism of pneumomediastinum, particularly in trauma, remains complex and multifactorial. The Macklin effect, characterized by alveolar rupture and air dissecting along the peribronchovascular sheaths, is one established explanation. Alternatively, air may migrate directly from the retroperitoneum through fascial planes, especially in cases involving retroperitoneal injury or elevated intra-abdominal pressure. Another theory involves the foramina of Morgagni and Bochdalek, diaphragmatic weak points that can serve as pathways for air passage from the peritoneum to the mediastinum [9].

Pneumorrhachis, the presence of air within the spinal canal, is another potential finding in such cases; however, it was not observed in our patient [11,12]. In instances of pneumothorax, such as in our case, it is postulated that a concurrent rupture of the parietal pleura may permit the migration of free pleural air into the mediastinal space. Concurring with Pavrey et al. [13], we hypothesize that the co-occurrence of pneumomediastinum, pneumoperitoneum, and pneumoretroperitoneum was likely due to the existing pneumothorax, exacerbated by the high settings during mechanical ventilation secondary to increased intrathoracic pressure causing a rupture of the alveoli and air escaping [14].

Literature substantiates that such presentations often arise subsequent to interventions. For instance, Marwan et al. have documented a case where pneumomediastinum and pneumoperitoneum, as well as pneumoretroperitoneum, developed following colonic perforation after a diagnostic colonoscopy [15]. Similarly, Elkholy et al. have described a case where this dual presentation resulted from inadvertent tracheal tube misplacement [16]. These instances highlight the importance of thorough evaluation for hollow viscus injury. A precipitating history of a surgical procedure can hint toward an iatrogenic injury [13].

In managing such cases, we advocate for vigilant observation and conservative measures if isolated air is detected in CT. A systematic review of 339 patients suggests the limited rule of additional diagnostic tests like gastrografin swallow, bronchoscopy, and upper gastrointestinal endoscopy in the evaluation of patients with SPM [7].

## Conclusions

The patient presented with pneumomediastinum, pneumothorax, pneumoperitoneum, and pneumoretroperitoneum, findings that could easily raise alarms for catastrophic injuries like esophageal rupture. It demonstrates the Macklin effect as a critical yet often underrecognized phenomenon in trauma care. Despite the alarming radiological findings, conservative management was sufficient for resolution without surgical intervention. Comprehensive imaging, adherence to trauma protocols, and multidisciplinary evaluation allowed accurate diagnosis and the subsequent decision for close observation. We reported this case to highlight the importance of careful clinical judgment to avoid unnecessary interventions and to emphasize the value of conservative management in selected trauma patients. We call for further studies to evaluate and clinically predict this phenomenon to better understand it and reduce unnecessary investigations and interventions.
